# The Glymphatic System and Neurosurgery: A Comprehensive Narrative Review of Current Concepts and Future Directions

**DOI:** 10.3390/jcm15145700

**Published:** 2026-07-21

**Authors:** Kadir Çetinkaya, Yaşar Ünsal

**Affiliations:** 1 Department of Neurosurgery, Hıtıt University Erol Olcok Training and Research Hospital, Çorum 19040, Türkiye; 2 Department of Neurosurgery, Bilkent City Hospital, Ankara 06800, Türkiye

**Keywords:** glymphatic system, aquaporin 4, neurosurgical interventions, cerebrospinal fluid, traumatic brain injury

## Abstract

The glymphatic system is a recently defined perivascular waste elimination pathway responsible for the efficient clearance of metabolic waste and neurotoxic proteins in the central nervous system. This system facilitates the entry of cerebrospinal fluid (CSF) into the brain parenchyma via arterial perivascular spaces and its interaction with interstitial fluid (ISF) via glial cell-associated aquaporin-4 (AQP4) channels. It functions particularly actively during sleep. Impairment of glymphatic flow contributes to nerve cell damage and neuroinflammation in various pathologies such as Alzheimer’s disease, Parkinson’s disease, traumatic brain injury, subarachnoid hemorrhage, and neurological tumors. In neurosurgical practice, surgical positioning, anesthesia regimen, and intracranial pressure changes play a decisive role in glymphatic function, and perioperative modulation of the system can affect postoperative recovery and cognitive outcomes. Today, non-invasive imaging techniques and molecular biological approaches are deepening our understanding of the functioning of the glymphatic system in humans, and this system is emerging as a potential target in the diagnosis and treatment of neurological diseases. This review comprehensively addresses the basic anatomical and physiological principles of the glymphatic system, its role in pathological processes, and its clinical significance in neurosurgical applications.

## 1. Introduction

The central nervous system (CNS) has long been considered devoid of a conventional lymphatic system, a belief that has fueled the mystery surrounding how the brain clears metabolic waste. This paradigm has been substantially altered by the discovery of the glymphatic system, a brain-wide perivascular network responsible for facilitating the clearance of interstitial solutes through cerebrospinal fluid (CSF) exchange mechanisms [1]. The glymphatic system is a glia-dependent pathway that promotes the movement of CSF into the brain parenchyma via astrocytic aquaporin-4 (AQP4) water channels and enables the clearance of metabolic waste through perivenous pathways [2].

The term “glymphatic” reflects the dual role of glial cells and its functional similarity to the peripheral lymphatic system. In this model, CSF enters the periarterial spaces, exchanges with interstitial fluid (ISF) within the parenchyma, and exits through perivenous pathways, ultimately draining into the cervical lymph nodes [3]. This exchange is primarily driven by arterial pulsatility and AQP4 channel activity [4]. Notably, the glymphatic system is most active during sleep, particularly during non-rapid eye movement (NREM) sleep, when the interstitial space expands by approximately 60%, allowing enhanced convective clearance of metabolites such as amyloid-β and tau proteins, which are implicated in neurodegenerative diseases [5].

Impairment of glymphatic flow has been implicated in a variety of neurological disorders. In Alzheimer’s disease (AD), glymphatic dysfunction may contribute to the accumulation of neurotoxic proteins such as amyloid-β and phosphorylated tau, thereby exacerbating synaptic loss and cognitive decline [6]. Similarly, in Parkinson’s disease, impaired CSF-mediated clearance may play a role in α-synuclein aggregation [7]. In addition, recent studies have demonstrated that traumatic brain injury (TBI), subarachnoid hemorrhage (SAH), and ischemic stroke are associated with reduced glymphatic activity, contributing to cerebral edema and secondary injury [8,9].

The significance of the glymphatic system extends beyond chronic neurological disorders and into neurosurgical practice. Various surgical and perioperative factors, including head positioning, anesthetic regimens, intracranial pressure fluctuations, and surgical manipulation, may substantially influence glymphatic flow. For example, the lateral decubitus position has been shown to enhance glymphatic transport compared with prone or upright positions, and is often considered optimal during sleep. While supine positioning also offers benefits over prone, the lateral position appears to be superior for waste clearance [10]. Furthermore, the choice of anesthesia has important implications: ketamine/xylazine anesthesia enhances glymphatic transport, whereas isoflurane may impair it [11].

Perioperative modulation of the glymphatic system has important implications. Efficient clearance of metabolic waste products and inflammatory mediators may influence postoperative recovery, cognitive outcomes, and the efficacy of intrathecal drug delivery [12]. Furthermore, the loss of aquaporin-4 polarization, which is observed during aging and in certain pathological conditions, may disrupt glymphatic flow, thereby increasing susceptibility to postoperative complications and delaying neurological recovery [4].

Despite the growing body of evidence, the clinical translation of glymphatic research remains in its early stages. The vast majority of studies have been conducted in animal models, and the noninvasive assessment of glymphatic function in humans continues to represent a major technical challenge. Nevertheless, advances in imaging techniques, including contrast-enhanced magnetic resonance imaging (MRI) and diffusion tensor imaging along the perivascular space (DTI-ALPS), have enabled the preliminary visualization of glymphatic pathways in the human brain [13].

While the glymphatic system has been extensively reviewed in the context of neurodegenerative diseases and basic physiology, its specific implications for neurosurgical practice remain underexplored. This review distinguishes itself by providing a dedicated, neurosurgery-focused analysis. Specifically, it is among the first to comprehensively evaluate the impact of perioperative factors—such as surgical positioning and anesthetic regimens—on glymphatic function. Furthermore, this review uniquely integrates recent human clinical data utilizing the DTI-ALPS index to predict surgical outcomes, and it critically examines the physiological rationale behind emerging surgical techniques like cisternostomy through the lens of glymphatic and meningeal lymphatic drainage. By bridging basic science with practical neurosurgical considerations, this manuscript aims to provide actionable insights for clinicians and identify specific targets for future translational research. It is important to note that the majority of mechanistic insights regarding the glymphatic system, including the effects of surgical positioning and anesthesia, are currently derived from rodent models, and their direct translation to human clinical practice requires further validation.

## 2. Literature Search Strategy

This article is structured as a comprehensive narrative review. To identify relevant literature, a comprehensive search was conducted across the PubMed and Scopus databases for articles published between 1 January 2012, and 30 June 2026. The search strategy employed a combination of keywords and Boolean operators, including but not limited to: (“glymphatic system” OR “glymphatic pathway”) AND (“neurosurgery” OR “neurosurgical” OR “traumatic brain injury” OR “TBI” OR “subarachnoid hemorrhage” OR “SAH” OR “intracerebral hemorrhage” OR “ICH” OR “hydrocephalus” OR “normal pressure hydrocephalus” OR “NPH” OR “brain tumor” OR “neuro-oncology” OR “decompressive craniectomy” OR “cisternostomy” OR “anesthesia” OR “surgical positioning”). The authors reviewed titles and abstracts to select peer-reviewed original investigations and high-impact review articles that provided fundamental mechanistic insights or demonstrated clear relevance to neurosurgical practice. Both animal and human studies were included to provide a broad overview of the field. Articles published in languages other than English or classified as gray literature (e.g., unpublished theses, un-peer-reviewed conference abstracts) were generally excluded. However, publicly available clinical trial protocols and updates from reputable registries were considered for contextual information regarding ongoing neurosurgical research, though not formally cited as primary evidence. The selected literature was then synthesized to construct a narrative framework detailing the anatomical, physiological, and clinical aspects of the glymphatic system in neurosurgery.

### 2.1. Anatomy and Physiology of the Glymphatic System

**Figure 1:**
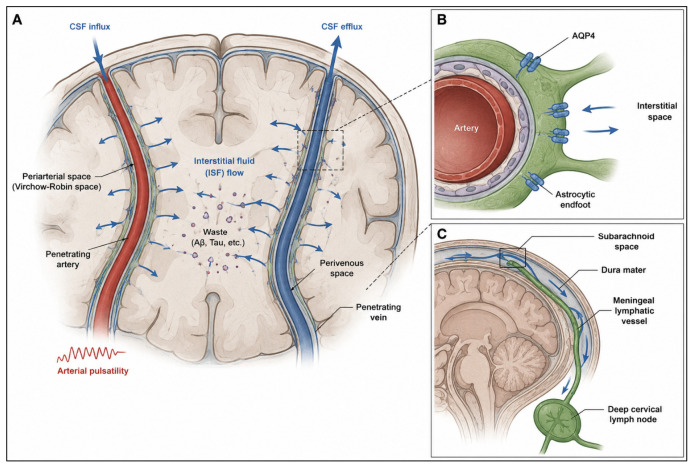
Comprehensive Schematic of the Glymphatic System Pathway. (**A**) Overview of fluid dynamics: Cerebrospinal fluid (CSF) enters the brain parenchyma via the periarterial spaces (Virchow-Robin spaces) surrounding penetrating arteries, driven by arterial pulsatility. Directional arrows indicate the convective flow of CSF mixing with interstitial fluid (ISF) through the extracellular space, facilitating the clearance of metabolic waste products (e.g., amyloid-β, tau). The fluid and solutes then exit via perivenous spaces. (**B**) Inset detailing the neurovascular unit: Highlighting the critical role of astrocytic endfeet, which densely express polarized Aquaporin-4 (AQP4) water channels that regulate the influx and efflux of fluid between the perivascular spaces and the interstitium. (**C**) Connection to lymphatic drainage: Illustrating the efflux pathways where perivenous fluid reaches the subarachnoid space and subsequently drains into the meningeal lymphatic vessels located within the dura mater, ultimately emptying into the deep cervical lymph nodes.

The glymphatic system is a recently discovered waste clearance pathway that maintains brain homeostasis in the central nervous system (CNS) through the integration of cerebrospinal fluid (CSF) and interstitial fluid (ISF) dynamics [1]. First described in mice by Iliff et al. in 2012 using real-time two-photon imaging [1], this system has altered existing paradigms in neurophysiology by revealing a highly organized and active solute clearance mechanism that is functionally analogous to the peripheral lymphatic system [2] (Figure 1).

### 2.2. Perivascular Entry and CSF Distribution

CSF enters the brain primarily through periarterial spaces surrounding penetrating arteries, in a process largely driven by arterial pulsatility [14]. These perivascular channels, historically referred to as Virchow–Robin spaces, serve as low-resistance conduits that allow CSF to penetrate into the brain parenchyma. Advanced imaging techniques, including dynamic contrast-enhanced MRI and fluorescent tracer studies, have confirmed that CSF flows in a directed and organized manner from the subarachnoid space toward deeper cortical and subcortical regions [15].

Tracer studies in animal models have demonstrated that this periarterial CSF influx distributes fluid deep into the brain tissue, particularly along the territories of the middle cerebral arteries [1]. The pressure gradients required for this flow are thought to arise from the combined effects of cardiac systolic pulsations, vasomotion, respiratory mechanics, and potentially glial cell activity [16]. This highly dynamic structure contrasts with the historically passive view of CSF circulation, in which flow was considered to be driven solely by pressure differences between the ventricles and the subarachnoid space.

### 2.3. Convective Flow Through the Interstitial Space

After entering the brain parenchyma, CSF mixes with ISF and facilitates the movement of solutes through bulk convective flow. This process differs from simple diffusion and enables the rapid clearance of large molecules, including neurotoxic proteins such as amyloid-β and tau [17]. Clearance primarily occurs along perivenous pathways, eventually draining into the subarachnoid space, meningeal lymphatic vessels, and ultimately into the systemic lymphatic circulation through the deep cervical lymph nodes [17].

Interestingly, this convective flow is not uniform throughout the brain. Regional variations have been observed, with structures such as the hippocampus and basal ganglia exhibiting higher glymphatic activity, likely reflecting their dense vascularization and astrocytic architecture [18]. Furthermore, the preferential directionality of glymphatic flow suggests a tightly regulated system influenced by the underlying neurovascular organization.

While the concept of bulk convective flow is central to the glymphatic hypothesis, it is important to acknowledge the ongoing controversy regarding the exact physical mechanisms of solute transport. Smith and Verkman [19] have critically challenged the glymphatic model, arguing based on theoretical modeling and experimental data that bulk convective flow through the narrow and tortuous extracellular space is physically implausible, and that diffusion alone is sufficient to account for solute movement in the brain parenchyma. Proponents of the convective model argue that arterial pulsatility and the dynamic expansion of the interstitial space during sleep create sufficient pressure gradients to drive bulk flow. The interpretation of AQP4 knockout data also remains a point of contention; while the original glymphatic studies attribute the reduced clearance in AQP4-deficient mice to impaired convective flow, critics suggest that AQP4 deletion may alter the extracellular space volume or tortuosity, thereby affecting diffusion rather than convection. A balanced understanding of these opposing viewpoints is crucial, as the true mechanism may involve a complex interplay of both diffusive and convective processes depending on the physiological state and anatomical region.

### 2.4. Astrocytes and Aquaporin-4 Channels: A Central Role

The astrocytic endfeet surrounding cerebral blood vessels express high densities of aquaporin-4 (AQP4) water channels, which are essential for facilitating transmembrane water transport between the CSF and ISF compartments. Mice lacking the AQP4 gene exhibit a 70–90% reduction in glymphatic transport efficiency, demonstrating the central role of this protein in system functionality [19]. Furthermore, the polarized distribution of AQP4 within astrocytic endfeet is crucial for maintaining the directional flow of water and solutes; loss of this polarization, as observed with aging or following brain injury, significantly impairs clearance [20].

Astrocytes not only facilitate water movement but also participate in neurovascular coupling, metabolic regulation, and extracellular ion buffering, functions that are closely integrated with glymphatic transport. Recent transcriptomic analyses have demonstrated that astrocytic genes regulating AQP4 expression are among the most dynamically altered genes in conditions such as Alzheimer’s disease and traumatic brain injury [21].

### 2.5. Outflow Pathways and Lymphatic Integration

Following interstitial transport, solutes and waste products exit the brain through perivenous spaces and accumulate in the subarachnoid CSF. From there, a substantial proportion is drained through meningeal lymphatic vessels lining the dura mater adjacent to the sagittal and transverse sinuses [22]. These vessels have been recently identified in mammals and have been shown to directly connect with the cervical lymph nodes, providing a critical immune interface for central nervous system (CNS) surveillance [23].

Disruption of these lymphatic structures has been implicated in various neurological disorders. For example, Louveau et al. demonstrated that ablation of meningeal lymphatics in Alzheimer’s disease models exacerbated amyloid pathology [17]. The functional integration between glymphatic influx and lymphatic efflux constitutes what some researchers now refer to as the “glymphatic–lymphatic” continuum.

### 2.6. Developmental and Pathological Changes

The glymphatic system exhibits an age-related decline, with reductions in AQP4 expression, arterial compliance, and perivascular integrity observed in aged mice and humans [6,20]. This decline may partially explain the increased burden of neurodegenerative diseases in older populations and the poorer recovery profiles following neurosurgical interventions.

In neonates and pediatric patients, glymphatic flow appears to be more robust; however, this remains an active area of investigation. Pathological conditions such as traumatic brain injury, stroke, subarachnoid hemorrhage, and brain tumors have been shown to impair glymphatic function, primarily through inflammation-induced astrogliosis and disruption of perivascular architecture [24].

### 2.7. Glymphatic Dysfunction in Common Neurosurgical Conditions

Glymphatic clearance plays a critical role in maintaining homeostasis within the central nervous system (CNS). Glymphatic dysfunction has been recognized as an important contributor to various neurosurgical pathologies, including traumatic brain injury (TBI), subarachnoid hemorrhage (SAH), intracerebral hemorrhage (ICH), hydrocephalus, and brain tumors [25]. Experimental and clinical studies have demonstrated that disruption of aquaporin-4 (AQP4) polarization or perivascular flow leads to the accumulation of metabolic waste products, increased interstitial fluid pressure, and neuroinflammation [26] (Table 1).

### 2.8. Traumatic Brain Injury

Traumatic brain injury (TBI) remains a major cause of morbidity and mortality worldwide [35]. Given the substantial morbidity and mortality associated with these injuries, there is considerable interest in optimizing treatment strategies. In TBI, glymphatic impairment is often a rapid secondary consequence of the initial injury, driven by astrocytic swelling and cytotoxic edema. However, this secondary dysfunction then becomes a primary exacerbating factor, contributing to the accumulation of neurotoxic proteins and inflammatory mediators, thereby worsening secondary brain injury and hindering recovery. In rodent models of TBI, studies have demonstrated that glymphatic transport is acutely impaired due to astrocytic swelling, cytotoxic edema, and AQP4 depolarization, resulting in reduced clearance of tau proteins and other neurotoxic solutes [26]. The Monro–Kellie doctrine explains that, due to the rigid structure of the skull, there is only a fixed volume capacity for the three major intracranial components: brain parenchyma, blood, and cerebrospinal fluid (CSF) [36]. Swelling causes a significant disruption in the delicate balance among these three components, potentially resulting in compression of the brain parenchyma and direct injury or ischemia due to reduced cerebral perfusion. When medical management is insufficient to control intracranial pressure (ICP), surgical options such as external ventricular drainage, craniotomy, or decompressive craniectomy are performed [37]. It is crucial to note that while the Monro–Kellie doctrine is fundamental to understanding intracranial dynamics in an intact skull, its applicability changes significantly after decompressive craniectomy. Once the skull is opened, the intracranial compartment is no longer a fixed-volume rigid container, and the doctrine, in its strict sense, ceases to apply.

The findings of the Randomized Evaluation of Surgery with Craniectomy for Uncontrollable Intracranial Pressure (RESCUEicp) trial showed that decompressive craniectomy, including hemicraniectomy and bifrontal decompression, was associated with improved survival compared with continued maximal medical management. However, this survival advantage was not accompanied by a significant increase in favorable functional outcomes, underscoring the need for more effective surgical strategies for the management of severe intracranial hypertension [38]. Beyond decompressive craniectomy, CSF diversion is another established strategy for lowering ICP by reducing intracranial volume and creating additional compensatory space within the cranial cavity [39]. External ventricular drainage remains the most widely used technique for this purpose. However, increasing evidence suggests that ventricular CSF and interstitial fluid exchange differ from the communication between the basal cisterns and the brain parenchyma. Based on this concept, it has been proposed that impaired CSF absorption following traumatic brain injury may promote the movement of CSF from the basal cisterns into the cerebral interstitium. The resulting accumulation of interstitial fluid may contribute to the development and progression of cerebral edema under conditions of elevated intracranial pressure [40].

Based on these findings, it has been hypothesized that microsurgical opening of the basal cisterns with external drainage (cisternostomy) may improve outcomes in patients. In a study conducted by Chandra et al., a randomized controlled trial comparing cisternostomy and decompressive craniectomy was performed in 50 patients (25 in each group), demonstrating improved ICP control and some improved outcomes in selected patient populations [41]. Despite these encouraging preliminary findings, the available clinical evidence remains limited by small sample sizes and experience from only a few surgical centers. Consequently, larger multicenter studies are necessary to confirm the reproducibility, safety, and clinical effectiveness of this surgical approach.

Despite these encouraging preliminary findings, the available clinical evidence remains limited by small sample sizes and experience from only a few surgical centers. Consequently, larger multicenter studies are necessary to confirm the reproducibility, safety, and clinical effectiveness of this surgical approach. Additional indirect support for cisternal CSF drainage is provided by studies evaluating lumbar drainage in patients with TBI [42]. Because lumbar drains continuously remove CSF that communicates with the basal cisterns, they may contribute to intracranial pressure (ICP) control. Although concerns regarding the potential risk of transtentorial herniation have historically limited their use, recent systematic evidence indicates that lumbar CSF drainage can achieve a significant reduction in ICP in appropriately selected patients with TBI [43].

Experimental studies further demonstrate that TBI profoundly disrupts glymphatic function. Animal models have shown that astrocytic swelling, cytotoxic edema, and loss of normal AQP4 polarization impair perivascular fluid transport, thereby reducing the clearance of tau and other neurotoxic metabolites [44]. Beyond the glymphatic pathway, dysfunction of the meningeal lymphatic system also appears to contribute to secondary brain injury. In a mouse model, Bolte et al. [45] reported persistent impairment of meningeal lymphatic drainage for up to one month after TBI, accompanied by reduced clearance of CSF and macromolecules. Elevated ICP was proposed as a key factor contributing to this dysfunction, creating a vicious cycle in which impaired lymphatic outflow further compromises interstitial fluid clearance and aggravates intracranial hypertension [46].

### 2.9. Hydrocephalus

Hydrocephalus is a condition characterized by the abnormal accumulation of cerebrospinal fluid (CSF) within the cerebral ventricles, resulting in increased intracranial pressure [47]. In idiopathic normal pressure hydrocephalus (iNPH), glymphatic dysfunction is increasingly recognized as a primary pathogenic factor, leading to reduced CSF clearance and accumulation of neurotoxic substances, which in turn contributes to ventricular enlargement and cognitive decline. This suggests that glymphatic impairment is not merely an associated phenomenon but a key driver of disease progression in iNPH [13,48]. Studies suggest that glymphatic dysfunction is a fundamental component of iNPH pathophysiology and may also be associated with poor response to shunt treatment [6]. Dysfunction or mislocalization of AQP4 water channels may disrupt the movement of CSF from perivascular spaces into the brain parenchyma, thereby influencing the development of hydrocephalus [49]. Therefore, targeting glymphatic function represents a potential therapeutic strategy for improving treatment outcomes in patients with iNPH.

### 2.10. Subarachnoid Hemorrhage

Following SAH, glymphatic impairment is initially a direct consequence of physical obstruction by extravasated blood and subsequent inflammation. However, this impairment rapidly transitions into a critical secondary driver of complications such as hydrocephalus and delayed cerebral ischemia, by hindering the clearance of toxic blood products and inflammatory mediators. Following subarachnoid hemorrhage (SAH), blood products within the subarachnoid space disrupt perivascular fluid pathways by obstructing normal cerebrospinal fluid (CSF) circulation and restricting interstitial fluid clearance [31]. These experimental observations are supported by recent intrathecal contrast-enhanced MRI studies demonstrating delayed glymphatic transport and reduced clearance after SAH [27,28,50]. Compelling evidence indicates that dissolving fibrin clots within the subarachnoid space via intrathecal tissue plasminogen activator (tPA) significantly restores cerebrospinal fluid (CSF) dynamics [51]. These collective insights strongly reinforce the premise that fibrin accumulation within glymphatic networks obstructs CSF resorption, thereby driving the pathogenesis of post-hemorrhagic hydrocephalus. Contemporary neurovascular research heavily emphasizes the pivotal role of aquaporin water channels in regulating cerebrospinal fluid (CSF) homeostasis, particularly regarding the disruptive alterations in fluid dynamics observed following SAH [52]. Within this molecular family, aquaporin-4 (AQP4) stands out as an indispensable mediator that drives the bidirectional exchange between CSF and interstitial fluid (ISF), serving as a core structural determinant necessary for maintaining physiological glymphatic clearance [53].

Preclinical data demonstrates that the compromise of AQP4 channels modulates the transit of blood-borne macromolecules across perivascular spaces after subarachnoid hemorrhage, which subsequently reshapes glymphatic kinetics within the compromised cerebral tissue [29]. Nonetheless, alterations in this AQP4-mediated fluid dynamics do not linearly correlate with enhanced neurological outcomes; this discrepancy indicates that clinical recovery following SAH is governed by a multifaceted cascade that extends well beyond isolated glymphatic failure, encompassing intertwined pathways such as robust neuroinflammation, microvascular blood–brain barrier disintegration, and secondary neurodegenerative cascades [54]. This seemingly paradoxical finding highlights the complex, dual-phase role of AQP4 in SAH pathophysiology. In the acute phase post-SAH, AQP4 can facilitate the influx of toxic blood components and inflammatory mediators into the brain parenchyma, contributing to early cytotoxic edema and secondary injury. However, in the subacute and chronic phases, AQP4 is crucial for the efficient clearance of metabolic waste products, including hemoglobin degradation products and neurotoxic proteins, which accumulate due to impaired glymphatic flow. Therefore, while AQP4 deficiency might mitigate early influx, it simultaneously compromises the long-term clearance mechanisms essential for neurological recovery, leading to a net neutral or even detrimental effect on overall outcome.

### 2.11. Oncology

In neuro-oncology, glymphatic dysfunction appears to be a complex interplay of primary and secondary factors. Tumor growth and associated peritumoral edema directly disrupt glymphatic architecture, making it a secondary consequence. Yet, this disruption then becomes a primary contributor to the worsening of peritumoral edema and potentially facilitates tumor cell dissemination (leptomeningeal metastasis), thus exacerbating disease progression. The role of the glymphatic system in cerebral oncological processes remains even more preliminary than in the conditions described above. In brain tumors such as gliomas or metastases, abnormal vascularization, disruption of the blood–brain barrier, and peritumoral edema alter the normal architecture of the glymphatic system [55]. Impaired perivascular flow has been demonstrated in tumor-bearing mice, suggesting disrupted interstitial clearance in the brain parenchyma adjacent to the tumor [32]. Interestingly, a strong correlation exists between peritumoral edema and upregulation of AQP4, suggesting that at least a proportion of the associated edema in intracranial oncological lesions may be related to glymphatic system dysfunction [56] (Table 2).

Recent studies, such as those by Scalia et al. [32] and Jia et al. [33], have further elucidated this relationship by demonstrating that the loss of AQP4 polarization at the astrocytic endfeet surrounding tumors exacerbates the accumulation of interstitial fluid, thereby worsening peritumoral edema and increasing local tissue pressure. This elevated pressure further compresses perivascular spaces, creating a vicious cycle of impaired clearance and progressive edema. These dysfunctions are important not only for understanding peritumoral edema but also for predicting drug distribution and therapeutic resistance. The principles of the glymphatic system are increasingly being leveraged to improve targeted therapies. Convection-enhanced delivery (CED), for instance, utilizes continuous positive pressure to drive therapeutic agents directly into the brain parenchyma, effectively bypassing the blood–brain barrier. By understanding and potentially modulating glymphatic flow pathways, CED can be optimized to achieve wider and more uniform distribution of chemotherapeutic agents within the tumor bed and surrounding infiltrated tissue.

The mechanisms underlying the establishment of metastatic tumors within the brain remain incompletely understood and are an area of ongoing research. Emerging evidence suggests that, in addition to the cerebral vasculature, cerebrospinal fluid (CSF) circulation and perivascular pathways may influence tumor dissemination within the central nervous system [57]. While definitive clinical data intersecting glymphatic transport with cerebral oncogenesis remains sparse, these perivascular networks are increasingly recognized as potential conduits for malignant cell dissemination, especially during leptomeningeal progression [58]; crucially, the physical occlusion of these same glymphatic and meningeal lymphatic channels by infiltrating neoplastic cells severely restricts cerebrospinal fluid (CSF) drainage, thereby precipitating communicating hydrocephalus—a frequent and morbid secondary manifestation of leptomeningeal malignancy [59].

### 2.12. Effects of Neurosurgical Interventions on the Glymphatic System

Surgical interventions may exacerbate or improve glymphatic dysfunction depending on the underlying pathology and the procedure performed. For example, decompressive craniectomy performed in traumatic brain injury (TBI) or ischemia may partially reduce intracranial pressure; however, it may also disrupt the pulsatile flow required for glymphatic clearance [26,27]. This disruption occurs because opening the skull significantly alters intracranial compliance. The arterial pulsations, which are a primary driving force for glymphatic flow, rely on the closed, rigid intracranial vault to generate a pressure wave that propagates through the perivascular spaces. When the skull is opened, this pulse pressure wave is attenuated, leading to a substantial reduction in the convective forces necessary for efficient CSF and ISF exchange. Consequently, while decompressive craniectomy effectively reduces bulk ICP, it can paradoxically impair glymphatic function by diminishing the pulsatile energy transfer. Conversely, resection of obstructive lesions (e.g., tumors or hematomas) may decompress perivascular spaces, normalize AQP4 expression, and restore glymphatic function [33,60].

Changes in intracranial compliance and cerebrovascular pulsatility resulting from craniotomy can influence CSF dynamics. Animal studies have demonstrated that dural opening and surgical trauma may transiently reduce glymphatic flow, particularly in the presence of postoperative inflammation [2,61]. Furthermore, external ventricular drainage and shunt placement in hydrocephalus have shown mixed effects; although they reduce ventricular pressure, they may not fully restore perivascular CSF transport, particularly in normal pressure hydrocephalus (NPH) [30]. Importantly, recent MRI-based investigations have examined pre- and postoperative glymphatic activity using the diffusion tensor imaging along the perivascular space (DTI-ALPS) index. Surgical decompression for Chiari malformation or tumor resection has been associated with improvements in the ALPS index and clinical recovery, suggesting a potential role for glymphatic measurements in outcome prediction [62,63].

### 2.13. Effects on Anesthesia and Postoperative Recovery

Anesthetic agents commonly used in neurosurgical procedures may significantly influence glymphatic activity. For example, certain general anesthetics, including dexmedetomidine and ketamine/xylazine, have been shown to enhance glymphatic flow, whereas others, such as isoflurane, may impair clearance by suppressing slow-wave sleep and reducing cerebrovascular pulsatility [12,64]. The choice of anesthetic technique, particularly favoring local/regional anesthesia and conscious sedation over general anesthesia, is crucial given the glymphatic system’s role in waste clearance. While empirical glymphatic data in humans remains limited, conscious sedation techniques (e.g., using dexmedetomidine) may provide a theoretical clinical parallel to sleep-like physiology, potentially preserving arterial pulsatility and glymphatic function more effectively than deep general anesthesia [65]. This approach may mitigate the negative impact of general anesthesia on glymphatic clearance, potentially leading to improved postoperative cognitive outcomes. This has critical implications for anesthesia management during prolonged surgical procedures, particularly in older adults, in whom glymphatic activity is already reduced [66].

**Figure 2:**
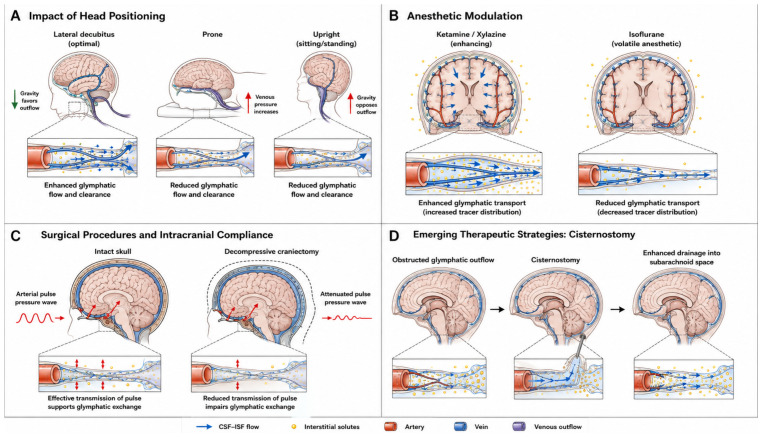
Perioperative and Surgical Modulation of Glymphatic Function. (**A**) Impact of Head Positioning: Schematic representation of how gravitational forces and venous outflow resistance influence glymphatic transport. The lateral decubitus position is depicted as optimal for maximizing clearance, while prone and upright positions are shown to increase venous pressure and potentially impede perivascular flow. (**B**) Anesthetic Modulation: Comparative illustration of anesthetic agents on glymphatic efficiency. Ketamine/xylazine is highlighted as an enhancer of glymphatic transport (represented by increased tracer distribution), whereas volatile anesthetics like isoflurane are shown to diminish flow. (**C**) Surgical Procedures and Intracranial Compliance: Illustrating the paradoxical effect of decompressive craniectomy. While effectively reducing bulk intracranial pressure (ICP), the opening of the skull (indicated by the dashed line) attenuates the arterial pulse pressure wave (red arrows), leading to a reduction in the convective forces required for efficient glymphatic exchange. (**D**) Emerging Therapeutic Strategies: Cisternostomy: Illustrating the surgical opening of the basal cisterns to facilitate the drainage of CSF and metabolic waste, thereby bypassing obstructed glymphatic pathways and reducing intracranial pressure in conditions like severe TBI.

Furthermore, postoperative delirium and cognitive dysfunction, particularly among elderly neurosurgical patients, may be partially attributed to impaired glymphatic clearance of neuroinflammatory mediators and metabolic waste products. Emerging evidence suggests that surgery- and anesthesia-induced neuroinflammation can disrupt glymphatic transport, potentially prolonging the retention of inflammatory cytokines within the central nervous system and contributing to perioperative neurocognitive disorders [67,68,69]. These findings highlight the potential of targeting glymphatic restoration in perioperative neuroprotective protocols (Figure 2).

### 2.14. The Glymphatic System as a Target for Therapeutic Modulation

Enhancement of glymphatic function holds therapeutic promise for neurosurgical patients. Investigated strategies include pharmacological AQP4 modulators, sleep optimization, respiratory and cardiovascular augmentation to enhance pulsatility, and position-based interventions such as the lateral decubitus sleep position [10,70]. CSF tracer studies and functional MRI assessments may help evaluate the efficacy of such interventions during the postoperative period.

Furthermore, the glymphatic system may represent a novel pathway for drug delivery, particularly for brain tumors and neuroinflammatory conditions. Advances in intrathecal nanoparticle-based delivery and convection-enhanced systems are being adapted to utilize glymphatic pathways to maximize drug penetration [34].

## 3. Conclusions

The glymphatic system has emerged as a promising area of neuroscience with important implications for neurosurgical diseases. Although much of the current evidence originates from experimental models, growing clinical data suggest that glymphatic dysfunction contributes to the pathophysiology of traumatic brain injury, subarachnoid hemorrhage, hydrocephalus, neurodegenerative diseases, and brain tumors. These findings highlight its potential as both a biomarker of disease progression and a therapeutic target.

Among the proposed therapeutic strategies, modulation of aquaporin-4 (AQP4) localization represents a particularly promising direction. However, a better understanding of the mechanisms regulating AQP4 polarization and its alterations in disease is required before targeted therapies can be translated into clinical practice. Likewise, surgical approaches aimed at preserving or restoring glymphatic function, including cisternostomy, remain investigational and require validation in well-designed clinical studies.

Overall, the glymphatic system represents an evolving field with considerable translational potential. Future research integrating experimental findings with high-quality clinical evidence will be essential to clarify its role in neurosurgical disorders and to determine whether modulation of glymphatic function can improve patient outcomes.

## 4. Strengths and Limitations

This review synthesizes current evidence on the glymphatic system and its relevance to neurosurgical diseases by critically integrating both experimental and clinical research. Its primary strength lies in bridging fundamental concepts of glymphatic physiology with their potential applications in disorders such as traumatic brain injury, subarachnoid hemorrhage, hydrocephalus, and intracranial tumors. In addition, recent advances in glymphatic assessment, including contrast-enhanced magnetic resonance imaging and diffusion tensor imaging analysis along the perivascular space (DTI-ALPS), together with discussion of perioperative factors such as anesthetic management and patient positioning, provide a clinically oriented perspective that may facilitate future translation into neurosurgical practice.

Nevertheless, several limitations should be acknowledged. The current body of evidence is characterized by substantial methodological variability, including differences in experimental models, study designs, evaluation techniques, and reported outcomes. This heterogeneity complicates direct comparisons across studies and limits the generalizability of the available findings. While we primarily focused on peer-reviewed literature, the exclusion of certain forms of gray literature, such as detailed clinical trial updates or protocols not yet published in full, might limit the inclusion of the most nascent clinical developments. In addition, the predominance of preclinical research in the existing literature limits the direct translation of many findings to clinical practice. Consequently, the applicability of experimental observations to human neurological diseases should be interpreted with appropriate caution until supported by robust clinical evidence. Furthermore, although imaging techniques such as DTI-ALPS have improved the evaluation of glymphatic function in humans, their ability to directly reflect actual glymphatic clearance remains uncertain and is a subject of ongoing debate. Furthermore, important questions remain regarding the mechanisms regulating AQP4 polarization and their potential therapeutic implications. Future well-designed, large-scale clinical investigations are essential to establish reliable glymphatic biomarkers and to clarify whether interventions aimed at modulating glymphatic function can translate into meaningful clinical benefits for patients with neurosurgical disorders.

## Figures and Tables

**Table 1:** Summary of Evidence for Major Clinical Claims Regarding the Glymphatic System in Neurosurgery.

Clinical Claim/Finding	Primary Evidence Source	Study Design/Model	Clinical Applicability	Key References
Lateral decubitus positioning is superior for glymphatic transport compared to supine, while both are more effective than prone or upright positions.	Animal (Rodent)	Experimental imaging studies	Requires human validation before altering surgical protocols.	[11,26]
Ketamine/xylazine enhances, while isoflurane impairs glymphatic flow.	Animal (Rodent)	Experimental imaging studies	May inform anesthetic choices, but human clinical trials are needed.	[11,27]
Impaired glymphatic clearance in TBI and SAH.	Mixed (Animal & Human)	Rodent models; Human MRI (DTI-ALPS, contrast-enhanced)	Strong translational potential; emerging as a diagnostic biomarker.	[26,28,29]
Cisternostomy improves outcomes by reducing ICP and edema.	Mixed (Animal & Human)	Rodent models; Early human clinical series	Promising, but requires large-scale randomized controlled trials.	[30,31]
DTI-ALPS index correlates with surgical outcomes in Chiari and tumors.	Human	Retrospective/Prospective clinical imaging studies	High potential for clinical use as a non-invasive prognostic tool.	[32,33,34]

**Table 2:** Glymphatic Dysfunction in Neurosurgical Conditions: Pathophysiological Role and Therapeutic Implications.

Condition	Primary Role of Glymphatic Dysfunction	Key Glymphatic Alterations	Therapeutic Rationale	Key References
Traumatic Brain Injury	Secondary consequence, then exacerbating factor	Astrocytic swelling, AQP4 depolarization, impaired waste clearance	Early restoration of flow to mitigate secondary injury; targeted clearance of neurotoxic proteins.	[26,37]
Hydrocephalus	Primary pathogenic factor	Reduced CSF clearance, AQP4 mislocalization, accumulation of neurotoxic substances	Enhance CSF flow, restore AQP4 polarization, improve waste removal.	[6]
Subarachnoid Hemorrhage	Direct consequence, then secondary driver of complications	Physical obstruction by blood, inflammation, impaired clearance of toxic products	Clearance of blood products, reduce inflammation, prevent secondary hydrocephalus.	[27,29]
Neuro-oncology	Secondary consequence, then primary contributor to edema/metastasis	Disrupted perivascular architecture, peritumoral edema, potential for tumor cell dissemination	Reduce peritumoral edema, optimize drug delivery (CED), inhibit metastatic spread.	[32,55,56]

## Data Availability

This review is based exclusively on previously published studies; therefore, no original datasets were generated or analyzed.

## Abbreviations

AD	Alzheimer’s Disease
AQP4	Aquaporin-4
BBB	Blood–Brain Barrier
CNS	Central Nervous System
CSF	Cerebrospinal Fluid
DTI-ALPS	Diffusion Tensor Imaging along the Perivascular Space
ICP	Intracranial Pressure
ICH	Intracerebral Hemorrhage
INPH	Idiopathic Normal Pressure Hydrocephalus
ISF	Interstitial Fluid
MRI	Magnetic Resonance Imaging
NPH	Normal Pressure Hydrocephalus
NREM	Non-Rapid Eye Movement
SAH	Subarachnoid Hemorrhage
TBI	Traumatic Brain Injury
tPA	Tissue Plasminogen Activator
